# Event and Comment

**Published:** 1906-07-15

**Authors:** 


THE
DENTAL REGISTER.

Vol. LX. July 15, 1906. No. 7.

### EVENT AND COMMENT.

### The San Francisco Situation.

As time goes on and we get more information in regard to the condition of the profession in San Francisco, the actual situation becomes more pitiful and seems to demand more sacrifices on the part of those of us who have had no such desperate experiences. The dental profession is probably more seriously affected than any other, for the reason that the large majority of offices were located in the burned districts, and the loss in every case was total, with little insurance to assist in re-establishing the office equipment. Then again the patients lost their possessions and many their business occupations, so that they have no earnings to pay for professional services, badly as they may need them. Of course, these conditions will right themselves in time, and the people will require the dentist's services and be able to compensate them. In the meantime, many dentists have despaired of ever being able to maintain themselves again in that place and have gone to other localities, while some have, per force, had to seek other means of gaining a living, others are striving to re-establish themselves in the best way that is practicable. Dr. Harry Carlton writes us that he has a bare room in Oakland which he hopes to equip as fast as practicable for serving what patients can find him. Dr. C. A. Devlin writes that he has gone over to Vallejo where he will start all over again.

The following extract from a recent letter will perhaps give us a good idea of what has befallen our brethren in San Francisco, as it is substantially the experience of most of them. He says: I lost everything I had in the world in the fire, I had a nice and select practice and was very happy and contended in my work. I dislike to recall the terrible disaster, as it was all so shocking that it gives one the feeling that it can never be forgotten. The scene of disaster with the terror stricken people was such that I shall never forget it. I am starting in a most humble way to re-establish my practice in this place where I formerly lived and shall hope for the best."

The dental relief committees in various parts of the country are doing well. So far about $10,000 have been raised and forwarded to Dr. J. S. Marshall, in San Francisco, who is doing what he can to help out the worst cases of need. It is hardly necessary for us to say that five times this amount should be raised and it will be none too much to assist the profession in re-establishing itself, and such a sum is none too much for the profession to contribute in view of our general prosperity and need of our suffering brethren. If every member of the profession would contribute two dollars or more, a handsome sum would be realized that would bring gladness and good cheer to the hearts of many who may be asking themselves whether after all life is worth living. The suggestion has been made that in every office there are unused instruments that could be sent to assist in at least temporarily equipping the devastated offices that would be put at once into glad hands for much needed services. Rents and office expenses are going to be high in San Francisco for a time, and fees will be few and small, and we doubt not that many dentists there will be glad to get appliances that are serviceable and out of use in our modernly equipped offices. Send any contributions you want to make to Dr. J. D. Patterson, Keith & Perry Building, Kansas City, Mo., and he will see that they get into good hands for distribution to those most needing them.

**Professional Men and Business Affairs.**

The statement is often made that dentists are notoriously bad business men, and it is some comfort to know that what we have been led to think was peculiar to dentists and clergymen finds a similar parallel in the physician's life. The following extract from an editorial in the *Medical World*, would indicate this fact and also to some extent account for it:

"Why the physician should be generally so incompetent in business affairs out of his own field of work is a question hard to answer offhand. His profession, perhaps, is so exacting that it leaves him little time to attend to other matters The work of a doctor, too, brings him into contact with so much misery and suffering, that it is apt to make him unwordly and trusting. Again, it is more easy to gain an audience with a physician than with members of other professions or business men. Be the reason, however, for the gullibility of the physician what it may, the fact remains that he is particularly open to the art of the fakir and swindler. There are signs that at last his eyes are being opened, and that in the future the doctor will lend deaf ear to the wiles and blandishments of the mining promoter and others of that ilk."

In the professions, to place absolute confidence in people is a fundamental article of the code. In fact, no devotee of any profession can make business methods and practices supreme without almost total loss of his self-respect, to say nothing of the loss of the confidence of his clients. A professional man who has his mind first fixed on making a monetary success of his life is a f ilure as a professional man, and can get little or no pleasure out of his life. It is, however, expected that professional men should pay their debts and save enough to keep them out of the poor house. The business shark who deliberately sets his trap to catch the savings of professional men is guilty of the same grade of crime as the contemptible villian who robs the widow and orphans or other confiding people. There is great need in in this country of some form of institution which shall be managed on an absolutely honest basis, which trusting people can patronize with confidence. Not that professional men are fools and idiots to be cared for by the government, but they have no time to spend from their important work in acquiring a training that will enable them to protect their business interests. A professional man who does his whole duty to his parish, has all the work that his time and strength will permit him to give to it. Insurance has been the main comfort of the professional man, but recent experiences teaches us that we can have slight assurance of honest dealing in such institutions. We may have to become the business agents of our own fortunes, but it certainly will be a distinct loss in professional spirit and efficiency.

